# Mitochondrial DNA sequences and transcriptomic profiles for elucidating the genetic underpinnings of cisplatin responsiveness in oral squamous cell carcinoma

**DOI:** 10.1186/s12863-022-01062-w

**Published:** 2022-06-21

**Authors:** Amnani Aminuddin, Pei Yuen Ng, Eng Wee Chua

**Affiliations:** Drug and Herbal Research Centre, Faculty of Pharmacy, Universiti Kebangsaan Malaysia, 50300 Kuala Lumpur, Malaysia

**Keywords:** Oral squamous cell carcinoma, Cisplatin response, Mitochondrial DNA, Oxford Nanopore Technologies, Gene expression, Human Clariom S array

## Abstract

**Objectives:**

Functional genetic variation plays an important role in predicting patients’ response to chemotherapeutic agents. A growing catalogue of mitochondrial DNA (mtDNA) alterations in various cancers point to their important roles in altering the drug responsiveness and survival of cancer cells. In this work, we report the mtDNA sequences, obtained using a nanopore sequencer that can directly sequence unamplified DNA, and the transcriptomes of oral squamous cell carcinoma (OSCC) cell lines with differing responses to cisplatin, to explore the interplay between mtDNA alterations, epigenetic regulation of gene expression, and cisplatin response in OSCC.

**Data description:**

Two human OSCC cell lines, namely H103 and SAS, and drug-resistant stem-like cells derived from SAS were used in this work. To validate our hypothesis that cisplatin sensitivity is linked to mtDNA changes, we sequenced their mtDNA using a nanopore sequencer, MinION. We also obtained the whole transcriptomic profiles of the cells from a microarray analysis. The mtDNA mutational and whole transcriptomic profiles that we provide can be used alongside other similar datasets to facilitate the identification of new markers of cisplatin sensitivity, and therefore the development of effective therapies for OSCC.

## Objective

Oral squamous cell carcinoma (OSCC) is a common malignant tumour of the head and neck [1]. To date, cisplatin remains the first-line chemotherapeutic agent for OSCC. However, its efficacy is limited by drug toxicity and the resistance capabilities of cancer cells [2]. Recently, mitochondrial DNA (mtDNA) abnormalities have been reported in various cancers, highlighting their immediate role in modulating cancer development and survival and therapeutic resistance [3, 4]. By altering mtDNA replication or transcription, mtDNA defects may impair mitochondrial functions, including energy production, biosynthesis, cell signalling, and regulation of oxidative stress and cell death [5–7]. In this work, we hypothesized that functional genetic variation in mtDNA could alter cisplatin-mitochondria interaction, potentially leading to enhanced toxicity or reduced drug efficacy.

**Table 1:** Overview of data files/datasets

Label	Name of data file/data set	File types (file extension)	Data repository and identifier (DOI or accession number)
Data file 1	Schematic overview of the study design	Image file (.tif)	Figshare (10.6084/m9.figshare.14701590) [9]
Data file 2	The general characteristics of the oral squamous cell carcinoma cell lines	Document file (.pdf)	Figshare (10.6084/m9.figshare.14701581) [10]
Data file 3	Details of sample processing and sequencing runs	Document file (.pdf)	Figshare (10.6084/m9.figshare.14703801.v1) [11]
Data file 4	Poretools visualizations of the FAST5 files generated by each sequencing run	Image file (.tif)	Figshare (10.6084/m9.figshare.14701572) [12]
Data file 5	Albacore base-called reads statistics generated using NanoStat	Document file (.pdf)	Figshare (10.6084/m9.figshare.14701578.v1) [13]
Data file 6	Mapping statistics generated using QualiMap and Geneious	Document file (.pdf)	Figshare (10.6084/m9.figshare.14701587.v1) [14]
Data file 7	The workflow for sequencing read processing and variant-calling analysis	Image file (.tif)	Figshare (10.6084/m9.figshare.14701584.v1) [15]
Data file 8	The transcriptomic profiles of SAS, SAS tumour spheres, and H103, as analysed via GeneChip Human Clariom S arrays	Image file (.tif)	Figshare (10.6084/m9.figshare.14701575.v1) [16]
Data set 1	Raw MinION sequencing data files	FAST5 file (.fast5)	Sequence Read Archive (Accession No.: PRJNA712949) [17]
Data set 2	Raw microarray data files	CEL file (.CEL)	Gene Expression Omnibus (Accession No.: GSE168424) [18]

In our previous work [8], we examined the influence of mtDNA alterations on the cisplatin responsiveness of human OSCC cell lines, SAS and H103, obtained from Japanese Cell Bank Research and European Collection of Authenticated Cell Cultures, respectively. We also derived cancer stem-like cells (CSCs) from the cell lines via a sphere-forming assay. We demonstrated that compared with SAS, H103 and the tumour spheres derived from SAS (which we loosely classified as a cell line) had reduced sensitivity towards cisplatin. To validate our prior hypothesis that cisplatin sensitivity is linked to mtDNA changes, we used MinION, a nanopore sequencer, to obtain the mtDNA profiles of the cells. We also performed a microarray-based transcriptomic analysis of the cells to explore the complex interplay between mtDNA and nuclear DNA, which could be manifested as genetic or epigenetic changes.

Here, we report the mtDNA sequences and the transcriptomes of the cells with differing responses to cisplatin [8]. One of the microarray datasets (H103), despite having been published elsewhere [8], has not been thoroughly analysed. Our findings add to the budding body of genomic and transcriptomic data, where pooled analyses may aid in the identification of molecular markers for predicting cisplatin response and enabling precise anticancer therapies of OSCC. The unique mechanism of nanopore sequencing, which draws on the distinctive electric current patterns produced by different DNA motifs, allows the detection of both sequence variations and DNA methylation. Therefore, the sequencing data can also be reused for in-depth analysis of mtDNA profiles and development of more effective tools for processing nanopore sequences.

## Data description

All the data files associated with this work are listed in Table 1. The study design is illustrated in Data file 1. The characteristics of the OSCC cell lines used in this work are described in Data file 2. The characterization of the stem cell-like tumour spheres and the measurements of cisplatin sensitivity of the three cell lines have been reported previously [8]. All the methods provided in the following sections are condensed versions of the methods described in our previous work [8].

### MinION sequencing

We performed six MinION sequencing runs for H103, SAS, and SAS tumour spheres using two MinION SpotOn Flow Cells version R9.5 (Oxford Nanopore Technologies (ONT), UK; Data file 3). We first co-extracted supercoiled mtDNA and nuclear DNA of the cells using QIAprep Miniprep Kit (QIAGEN, Germany) and Agencourt AMPure XP (Beckman Coulter Inc., USA) [19]. The sequencing libraries were prepared using the 1D Ligation Sequencing Kit (SQK-LSK108; ONT, UK), loaded onto the flow cells, and sequenced for 48 hours. The flow cells were washed using a Wash Kit (EXP-WSH002; ONT, UK) before they were reused for subsequent sequencing runs.

Raw sequencing signals stored in FAST5 files were acquired by MinKNOW version 1.6 (ONT, UK; Data set 1). The sequencing run performance was assessed using Poretools [20] (Data file 4). During sequencing, live base-calling with a read quality score threshold of 7 was executed by an in-built MinKNOW base-caller. To base-call all the reads, additional post-sequencing base-calling was performed using Albacore version 1.2.6 (ONT, UK). The quality of the base-called reads was assessed using NanoStat [21] (Data file 5). The base-called reads were mapped to the human reference genome assembly GRCh38 using BWA-MEM [22], generating alignment files (Sequence Alignment Map (SAM) format). The mapping statistics are provided in Data file 6 [23]. The SAM files were compressed into the binary format (BAM) using SAMtools [24]. The variants were called by Nanopolish [25], which compared the aligned reads with the revised Cambridge Reference Sequence of mtDNA in the GRCh38 assembly. The accuracy of variant calling was evaluated by a cross-check of the quality-filtered variants with Sanger sequencing, as described in our previous work [8]. The workflow for sequence reads processing and variant-calling analysis is provided in Data file 7.

### Microarray analysis

Total RNA was isolated and purified using innuPREP RNA Mini Kit (Analytik Jena, Germany) and RapidOut DNA Removal Kit (Thermo Fisher Scientific Inc., USA). The purified RNA samples were subjected to a whole transcriptomic analysis using the GeneChip Human Clariom S Array (Thermo Fisher Scientific Inc., USA; the analysis outsourced to Research Instruments Sdn. Bhd., Malaysia). The raw data files (CEL files) were obtained from the GeneChip Command Console Software (Thermo Fisher Scientific Inc., USA; Data set 2). The transcriptomic profiles of the cells, as described in Data file 8, were analysed using Transcriptome Analysis Console 4.0 (Affymetric Inc., USA). As reported previously, the findings of the microarray analysis were confirmed by real-time quantitative polymerase chain reactions (qPCR) [8].

## Limitations

The MinION sequencing produced raw signals stored in FAST5 files, whose size ranged from 321 MB to 6.94 GB (Data file 5). The notably varied file size was a consequence of variable sequencing output that was determined by the number of active nanopores in a flow cell at the start of a sequencing run. We found that the availability of active nanopores declined progressively after consecutive uses. Both amplicon and native DNA libraries of H103 that were sequenced on two used flow cells yielded low average depths of on-target coverage (Data file 6). As a result, Nanopolish could not call a complete profile of mtDNA variants for H103. Nevertheless, we performed ‘fill-in’ Sanger sequencing for regions that were not adequately covered to provide a complete set of mtDNA variants for H103, as described in our previous work [8].

All the nanopore reads had average quality scores consistently ≥10, computed from every position in the reads (Data file 4). The read quality scores may seem low if we assess them on the same scale used to interpret the widely used Phred scores; and if we consider the levels of data accuracy typically reported for other platforms. However, some have pointed out that the quality scores reflect error characteristics peculiar to MinION and should not be considered equivalent to the Phred-based scores [26]. Other researchers intending to reuse the data should be aware that the read quality scores may improve (or deteriorate) significantly with different base-calling schemes. Creating an algorithm for accurately rendering electrical signals derived from the nanopores into DNA sequences are still an area of ongoing research.

## Data Availability

The nanopore sequencing and transcriptomic data described in this Data Note were deposited in Sequence Read Archive (Accession No.: PRJNA712949) and Gene Expression Omnibus (Accession No.: GSE168424), respectively. The supplementary figures and tables can be accessed on Figshare. Please see Table 1 and references [9–18] for links to the relevant data repositories.

## Abbreviations

CSCs: Cancer stem-like cells
MtDNA: Mitochondrial DNA
OSCC: Oral squamous cell carcinoma
ONT: Oxford Nanopore Technologies
qPCR: Real-time quantitative polymerase chain reaction
SAM: Sequence Alignment Map
